# Biosynthesis and genome mining strategies for purine-derived *N*-nucleoside antibiotics

**DOI:** 10.3389/fmicb.2025.1684225

**Published:** 2025-11-12

**Authors:** Yujie Wu, ShiYu Wu, Xiaomin Niu, Xue Yu, Tuo Chen, Guangxiu Liu, Wei Zhang

**Affiliations:** 1 State Key Laboratory of Ecological Safety and Sustainable Development in Arid Land, Chinese Academy of Sciences, Lanzhou, Gansu, China; 2 Key Laboratory of Extreme Environmental Microbial Resources and Engineering, Lanzhou, Gansu, China; 3 State Key Laboratory of Cryospheric Science, Northwest Institute of Eco-Environment and Resources, Chinese Academy of Sciences, Lanzhou, Gansu, China; 4 University of Chinese Academy of Sciences, Beijing, China

**Keywords:** purine-derived *N*-nucleoside antibiotics, bioactivity, biosynthetic pathway, gene cluster mining, genome-guided discovery

## Abstract

**Background:**

The rise of antibiotic resistance underscores the urgent need for new antimicrobial agents. Nucleoside antibiotics are a structurally diverse class with broad biological activities, among which purine-derived *N*-nucleoside antibiotics (*N*-NAs) are of particular interest as their purine-linked frameworks enable diverse enzymatic modifications that yield compounds with distinct pharmacological profiles.

**Aim of the review:**

This review summarizes the bioactivity and biosynthetic logic of representative purine-derived *N*-NAs, including pentostatin-type compounds, angustmycins, and deazapurine analogues, to provide insights into the genome-based discovery of related natural products.

**Key scientific concepts of the review:**

By outlining conserved enzymes and genetic features within known BGCs, we illustrate how core enzyme probes can be used for genome-guided mining of putative clusters. This approach emphasizes both the opportunities and challenges in predicting novel *N*-NA producers from genomic data.

**Conclusion:**

Understanding the biosynthesis and genetic organization of *N*-NAs not only sheds light on their structural diversity but also provides a framework for genome mining. Specific subclasses such as pentostatin-, angustmycin-, and deazapurine-type compounds exhibit Structure–Activity relationships that could guide the rational design and genome-based discovery of new nucleoside antibiotics.

## 1 Introduction

Nucleoside antibiotics (NAs) comprise a diverse group of naturally occurring compounds derived from nucleosides or nucleotides, predominantly sourced from microorganisms. Given the pivotal role of nucleosides and nucleotides in fundamental metabolic processes, their antibiotic analogues demonstrate a broad spectrum of biological activities (Winn et al., 2010). Nucleoside antibiotics display antibacterial, antiviral, antifungal, antitumor, and herbicidal effects. Specifically, antibacterial NAs impede peptidoglycan synthesis by targeting bacterial cell wall biosynthesis (Winn et al., 2010). Antifungal NAs typically inhibit fungal chitin synthases or interfere with protein biosynthesis, while antiviral NAs hinder translation by blocking peptidyl transferase activity (Dmitriev et al., 2020; Zhou and Reynolds, 2024). Although these mechanisms are generally selective for each biological activity, certain NAs possess structural features that enable multiple modes of action, leading to overlapping antibacterial or antifungal effects (Niu and Tan, 2015).

Based on the relationship between sugars and nucleobases, these NAs can be classified into two distinct categories: *N*-nucleosides (C-N bond) and *C*-nucleosides (C-C bond) (Shiraishi and Kuzuyama, 2019). Based on the categorization of nucleoside groups, NAs can be classified into pyrimidine-derived and purine-derived analogues. Recent years have witnessed numerous reviews that comprehensively summarize the discovery and biosynthesis of pyrimidine-derived nucleoside antibiotics, while there is a scarcity of reports on purine-derived compounds (McErlean et al., 2021). Specifically, nucleoside antibiotics derived from guanine have received limited attention in the literature due to their infrequent discoveries. Structurally, these antibiotics can be further classified into four distinct classes: base analogues, simple nucleosides, acyl and glycosyl nucleosides, and nucleotides (Isono, 1988). Nucleoside analogues exhibit a remarkable chemical diversity, and their biosynthesis and biological activity are governed by tailoring enzymes.

Among NAs, purine-derived *N*-nucleoside antibiotics (*N*-NAs) form a distinctive subclass characterized by the attachment of purine bases to sugar moieties via N-glycosidic bonds. Purine-derived *N*-NAs have shown certain pharmacological advantages. For example, exogenous purine nucleosides such as guanosine have been reported to act as adjuvants enhancing the efficacy of β-lactam antibiotics against MRSA *in vitro* (Nolan Aaron et al., 2022). Furthermore, purine *N*-NAs analogues can combine potent antimicrobial activity with modifications that improve *in vivo* effect and reduce toxicity in some cases (Motter et al., 2024). These observations suggest that beyond scarcity of reports, the inherent bioactivity, structural versatility, and combinatorial potential make *N*-NAs promising candidates for antibiotic development.

**FIGURE 1:**
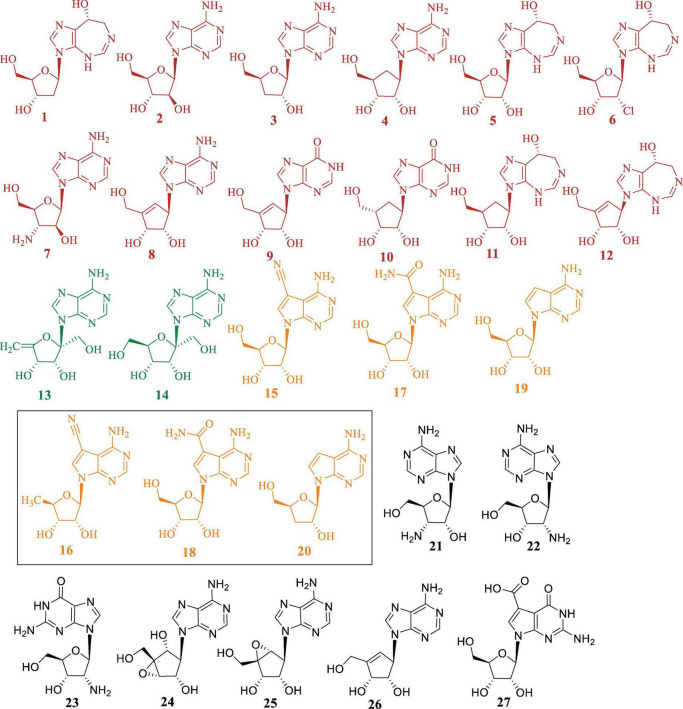
Chemical structures of representative purine-derived *N*-nucleoside antibiotics discussed in this review. Compounds are categorized and color-coded by structural class: red (**1-12**): pentostatin-related nucleosides, green (**13, 14**): angustmycins, orange (**15-20**): pyrrolopyrimidine nucleosides, black (**21-27**): other purine nucleoside analogs are discussed in detail in Section “2.4 *N*-NAs without Identified BGCs,” along with their names and corresponding references. The chemically synthesized analogues are highlighted with rectangular box.

In this review, we summarize the bioactivities and biosynthetic pathways of representative *N*-NAs (Figure 1), highlight common features of their biosynthetic gene clusters (BGCs), and discuss strategies for mining putative clusters from genomic databases. By integrating biosynthetic knowledge with genome-guided approaches, this review aims to provide a methodological framework that may accelerate the discovery of new nucleoside antibiotics.

## 2 Bioactivity and biosynthesis of purine-derived *N*-nucleoside antibiotics

### 2.1 Compounds originating from pentostatin-type BGCs

The compounds grouped in this section due to their similar biosynthetic origins from Pentostatin-type gene clusters. The compounds share a purine-derived core structure linked to a modified ribose or carbocyclic sugar moiety. These nucleoside analogues are typically characterized by variations at the 2′ or 3′ positions of the sugar and by modifications to the adenine or related purine base, such as deoxygenation, halogenation, or amination. The presence of carbocyclic or arabino-configured sugars confers enhanced metabolic stability and resistance to enzymatic degradation, features that are closely related to their potent biological activities.

Pentostatin (PTN, compound **1**), an effective inhibitor of adenosine deaminase, has gained significant utilization in the clinical management of malignant tumors and has garnered considerable interest among researchers (Figure 1). The distinctive 1,3-diazepine ring, particularly the *R* configuration of the chiral alcohol in the heterocyclic ring, endows it with distinctive biological activity and potent inhibitory properties against adenosine deaminase (Hanvey et al., 1987). As an anti-metabolic antitumor agent, **1** exerts its effects by elevating the levels of deoxyadenosine triphosphate in patients through the inhibition of adenosine deaminase activity in tumor cells. This action results in the suppression of ribonucleotide reductase, leading to a deficiency in the remaining three deoxynucleotide triphosphates essential for DNA synthesis. Consequently, the DNA synthesis process is impeded, thereby inhibiting lymphocyte proliferation (Schramm and Baker, 1985).

9-β-D-Arabinofuranosyladenine (Vidarabine, Ara-A, compound **2**), initially isolated from a marine sponge, exhibits significant efficacy as an antiviral agent against various viral DNA polymerases, making it a valuable therapeutic option for viral infections (Bergmann and Feeney, 1950). Furthermore, in their study, Awaya et al. (1979) successfully isolated **2** from *Streptomyces* and observed its potent herbicidal activity against *Echinochloa crus-galli*, *Digitaria adscendens*, and *Chenopodium ficifolium*.

Specifically, compounds 1 and 2 are produced by the same biosynthetic pathway, and similar biosynthetic relationships are observed for the pairs aristeromycin (ARM, 4) and coformycin (COF, 5), as well as 2′-Cl PTN (6) and 2′-amino deoxy-Ara (7) (Gao et al., 2017). Furthermore, neplanocin A (NEP-A, 8), neplanocin D (NEP-D, 9), carbocyclic inosine (10), carbocyclic COF (11) and adecypenol (12) are also considered PTN-related compounds (Zhang et al., 2020; Figure 1).

**FIGURE 2:**
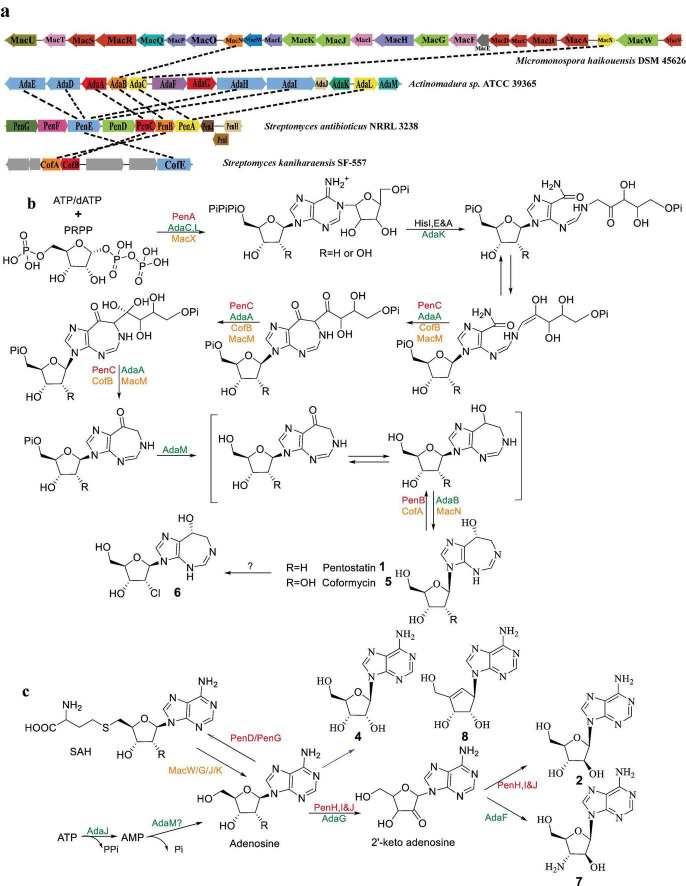
Proposed biosynthetic pathways of PTN-related compounds (Adapted from references: Wu et al., 2017; Xu et al., 2018; Gao et al., 2017). **(a)** PTN-like gene clusters (genes with the same color indicate enzymes with identical or similar functions), **(b)** biosynthetic pathway of compounds **1**, **5**, and **6**, and **(c)** biosynthetic pathway of compounds **2**, **4**, **7**, and **8**. Enzymes are color-coded by compound pairs: red for compounds **1** and **2**, orange for compounds **4** and **5**, and green for compounds **7** and **8**.

Notably, pentostatin-type BGC encodes two distinct product types through separate biosynthetic pathways. Despite being biosynthesized from the same gene cluster, these compounds exhibit complementary roles via a protector–protégé strategy. In this mechanism, compound **1** acts as a “protector” that mitigates potential cytotoxicity or metabolic interference caused by compound **2** (Wu et al., 2017). This strategy ensures that the biosynthetic production of both compounds can proceed efficiently within the same organism without self-toxicity, while maintaining high yields of both products. Figures 2a, b illustrate the distinct yet interconnected pathways, highlighting the specific enzymatic steps responsible for the biosynthesis of each compound.

The *pen* BGC, identified in *Streptomyces antibioticus* NRRL 3238, encodes the biosynthesis of both compounds **1** and **2**, which are synthesized through two distinct pathways originating from the same gene cluster. The *pen* cluster spans a length of 10.5 kb and encompasses a total of 10 genes, ranging from *penA* to *penJ*. Among them, PenA, PenB, and PenC play crucial roles as key enzymes in the biosynthesis of **1** (Figure 2a). PenA exhibits similarity to ATP phosphoribosyltransferase, specifically the HisG enzyme, which is responsible for the coupling of phosphoribosyl pyrophosphate (PRPP) to dATP in the histidine metabolic pathway. Furthermore, PenA has been identified as a rate-limiting enzyme in the biosynthetic pathway of **1** (Ren et al., 2020). PenB, a member of the short-chain dehydrogenase family, plays a crucial role in the final step in the biosynthesis of compound **1**. On the other hand, the precise function of PenC, which shares homology with phosphoribosylaminoimidazole-succinocarboxamide (SAICAR) synthase, remains unclear. The postulated function of Based on its homology, PenC is postulated to catalyze an amide- or C–N bond-forming step analogous to SAICAR synthase in purine biosynthesis, possibly linking an aminoimidazole intermediate to a ribose-phosphate derivative during the construction of the compound **1** scaffold. The remaining six enzymes, namely PenD-I, are associated with the synthesis of **2**. Among them, PenD and PenG function as S-adenosyl-L-homocysteine (SAH) hydrolases but with distinct roles. Specifically, PenG controls the forward reaction, while PenD is responsible for the reverse reaction, enabling the mutual conversion of SAH and adenosine. Furthermore, the biosynthesis of **2** involves the modification of three phytoene dehydrogenases (PenH-J) as a heteromeric complex. Finally, the major facilitator superfamily transporter encoded by *penE* is responsible for the transportation of compound **2** (Figure 2b).

The fungus *Cordyceps kyushuensis* has been found to possess a gene cluster, referred to as BGC of **1**, consisting of four genes (*ck1-ck4*), which play a crucial role in the biosynthesis of cordycepin (**3**) and **1** (Zhao et al., 2019). Similarly, in the fungi *Cordyceps militaris*, a cluster named *cns* has been identified, spanning a length of 10.3 kb and comprising four genes (*cns1* to *cns4*). Among these genes, *cns1* and *cns2* are responsible for the synthesis of **3**, while *cns3* and *cns4* are involved in the biosynthesis and transportation of compound **1** (Xia et al., 2017). Similar to the bacterial synthesis pathway, the protector-protégé strategy is also observed in fungus *Cordyceps kyushuensis*. Compound **1** exhibits the ability to protect **3** from deamination by adenosine deaminase, thereby enhancing the efficiency of **3** synthesis. The occurrence of similar *N*-NA biosynthetic architectures across bacteria and fungi likely reflects convergent recruitment of primary metabolic enzymes into specialized pathways, suggesting a functional rather than genealogical conservation.

The BGC *mac* responsible for the production of **4** and **5** pair in *Micromonospora haikouensis* DSM 45626 was identified through a BLASTP search in the NCBI database, with PenB and PenC as target enzymes (Xu et al., 2018). The cluster *mac* comprises 24 genes and spans a length of 25.3 kb. It was determined that the genes *macWGJK* are associated with the synthesis of **4**, while the genes *macXMNO* are involved in the synthesis of **5**. This confirms that the biosynthesis of **4** and **5** occurs through separate pathways. Furthermore, analysis of the metabolites of *M. haikouensis* using liquid chromatography-mass spectrometry (LC-MS) revealed the simultaneous detection of **8**, **9**, and **10**. Thus, the remaining genes in the cluster may related to these compounds. In *Streptomyces kaniharaensis* SF-557, a gene cluster known as *cof* was identified, which is responsible for the synthesis of compound **5**. This cluster bears a striking resemblance to the *pen* cluster (Figure 2; Ren et al., 2020).

In *Actinomadura* sp. ATCC 39365, the *ada* cluster was reported, which is responsible for the synthesis of the **6** and **7** pair (Gao et al., 2017). This cluster consists of 13 genes, *adaA-M*. Through bioinformatics analysis, it has been determined that *adaA-E*, adaF-J, and *adaLM* form a distinct transcription unit. The biosynthetic pathway of this gene cluster closely resembles that of the *pen* cluster (Figure 2). It is believed that *adaABCEKL* is involved in the biosynthesis of **6**, while the other four genes, *adaFGJM*, may play a role in the production of **7**.

### 2.2 Compounds originating from angustmycin-type BGCs

Angustmycins (AGMs) are a class of purine-derived nucleoside analogues featuring an exo-glycal moiety and a five-membered sugar ring. The two primary representatives of this class are angustmycin A (decoyinine, **13**) and angustmycin C (psicofuranine, **14**) (Yu et al., 2021). Compound **13** and **14** serve as analogues of adenosine, distinguished by an extra hydroxymethyl group at the C2′ position. The structural disparity between **13** and **14** lies in the presence of an exo-5,6-ene bond in the former (Figure 1).

These two compounds exhibit antibacterial and antitumor properties through the inhibition of GMP synthesis. Compound **13** additionally demonstrates the ability to inhibit spore formation in *Bacillus subtilis* and other bacteria (Hanka, 1960; Ochi, 1987). Research indicates that compound **13** effectively suppresses the invasion of melanoma cells *in vitro* and reduces tumorigenicity in immunocompromised mice, suggesting its potential as a novel anti-melanoma drug (Bianchi-Smiraglia et al., 2015). Furthermore, recent findings reveal that compound **13** acts as a cytokinin, known as linfusu, capable of inducing adventitious root or bud differentiation (Yu et al., 2021).

In characterizing the BGC encoding for the biosynthetic enzymes producing AGMs, Shiraishi et al. (2021) used conserved protein sequences such as Ari9 and MacI/T as probe. BLASTP searches was conducted on two *Streptomyces* strains, *S. angustmyceticus* NBRC 3934 and *S. decoyicus* NRRL 2666, both known to produce AGMs. This search identified *agm1* and *dcy1* in the respective genomes. Further examination of the flanking regions revealed that *agm1-6* and *dcy1-6* are associated with AGMs biosynthesis. Each cluster also contains a single transcriptional repressor gene: *agmR* in the *Streptomyces* strain harboring the *agm* cluster, and *dcyR* in the strain with the *dcy* cluster (Shiraishi et al., 2021).

**FIGURE 3:**
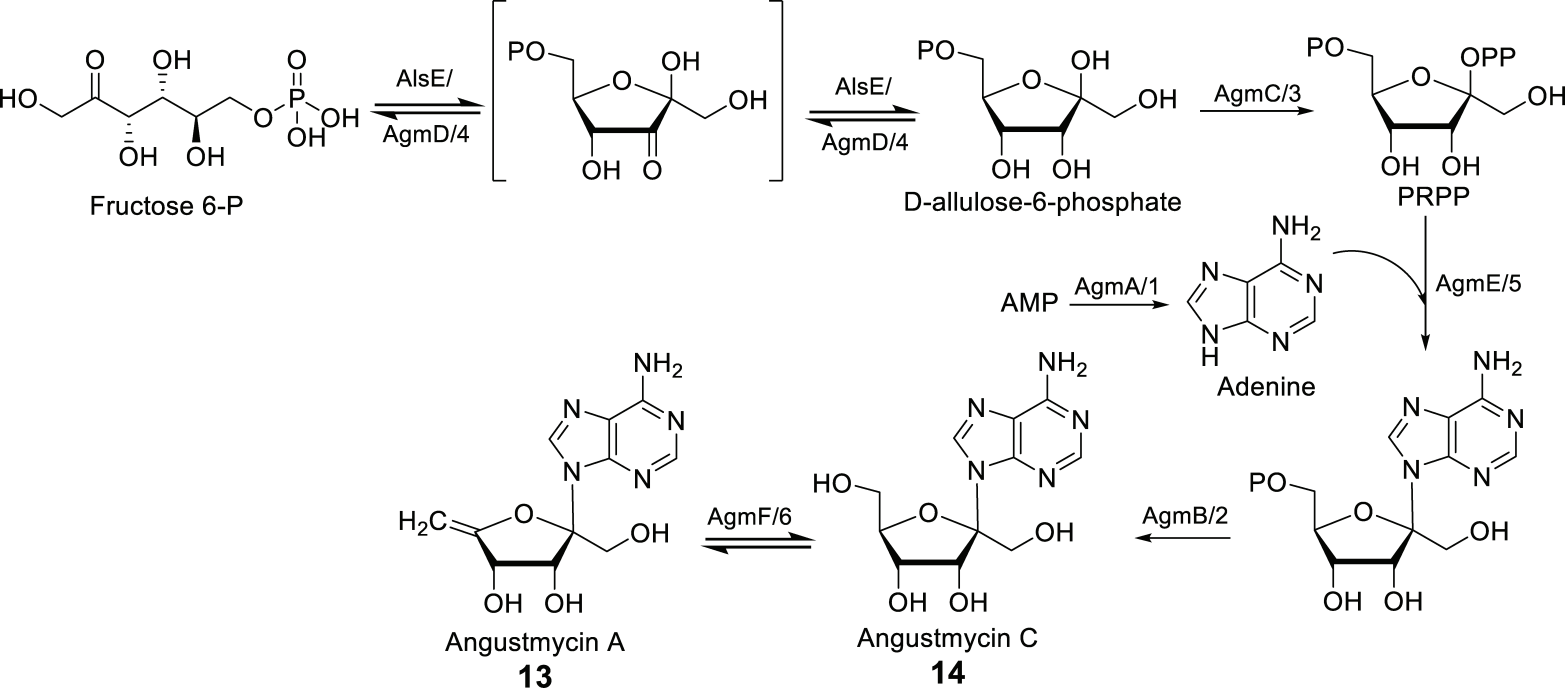
The biosynthesis pathways of AGMs [Adapted from references (Shiraishi et al., 2021; Yu et al., 2021)]. AgmA–F correspond to *S. angustmyceticus* JCM 4053, while agm1–6 correspond to *S. angustmyceticus* NBRC 3934. AMP is the abbreviation for adenosine monophosphate.

In the study conducted by Yu et al. (2021), the cluster *agm*, which is responsible for the synthesis of AGMs, was directly cloned from *S. angustmyceticus* JCM 4053. This cluster has a total length of 9.8 kb and consists of 9 genes, namely *agmA-E*, *agmR*, and *agmT1/T2*. The successful heterologous expression of the *agm* cluster was achieved in *Streptomyces coelicolor* M154, allowing for the analysis of the AGM synthesis pathway. The function of AgmA was determined to be AMP phosphoribohydrolase. *In vitro* experiments confirmed that AgmA utilizes AMP/dAMP as a substrate to produce adenine, thus enabling the synthesis of AGMs. AgmB has been demonstrated to possess AMP phosphatase activity, facilitating the conversion of AMP to adenosine. AgmC functions as a ribose 5-P pyrophosphokinase, catalyzing the conversion of ATP to AMP during the synthesis of AGMs. AgmD is presumed to exhibit a 3-epimerase function akin to AlsE, while AgmE acts as a phosphoallulosyltransferase, playing a role similar to APRTase in AGMs biosynthesis. Notably, a particularly intriguing step in the AGMs synthesis pathway involves the final dehydration reaction carried out by AgmF, resulting in the dehydration of **14** to yield **13**. When **14** is utilized as a substrate for the purpose of verifying the enzymatic reaction of AgmF, it is observed that the substrate undergoes incomplete transformation during the conversion to **13**. Consequently, it can be inferred that the dehydration reaction mediated by AgmF is reversible, as depicted in Figure 3 (Shiraishi et al., 2021; Yu et al., 2021).

In summary, the biosynthesis process of AGMs, as illustrated in Figure 3, involves the glycosyl epimerization (AgmD), followed by phosphorylation, adenine incorporation, dephosphorylation, and final dehydration (AgmF), yielding **13** from **14**.

### 2.3 Compounds originating from pyrrolopyrimidine-type BGCs

Pyrrolopyrimidine nucleosides analogues, commonly known as deazapurines, constitute an important class of compounds with remarkably diverse modifications. The distinctive feature of these compounds is the connection between the deazapurine core and the ribose moiety through *N*-glycosidic linkage. This review mainly summarizes the reported biological activities and biosynthetic pathways of deazapurine-containing compounds, namely toyocamycin (**15**), sangivamycin (**17**), tubercidin (**19**), along with analogues such as 5′-deoxy-toyocamycin (**16**), 5′-deoxy-sangivamycin (**18**), and 3′-deoxy-tubercidin (**20**) (Figure 1).

Compounds **15** and **17** were first identified and investigated in the 1970s (Uematsu and Suhadolnik, 1974). Compound **15** exhibits notable efficacy against diverse plant pathogenic fungi, thus presenting promising prospects for its utilization as an agricultural fungicide. On the other hand, compound **17** and its chemically modified derivatives hold considerable importance in clinical applications, demonstrating biological activities encompassing anti-tumor, anti-viral, and anti-bacterial properties (Uematsu and Suhadolnik, 1974, 1975; Zhang et al., 2022). Compounds **16** and **18**, derived through chemical total synthesis, represent dehydroxylated analogues of **15** and **17** (Dong et al., 2019).

Currently, three *Streptomyces* strains, namely *S. rimosus* ATCC 14673, *S. diastatochromogenes* 1628, and *S. ahygroscopicus* S91, have been reported to possess gene clusters associated with biosynthesis of **15** (McCarty and Bandarian, 2008; Xu et al., 2019; Ma et al., 2020; Liu et al., 2022; Zhang et al., 2022). Notably, the gene cluster in *S. rimosus* consists of 13 genes, denoted as *toyA-M*, responsible for the synthesis of both **15** and **17**. Conversely, the gene clusters in the other two strains, *toyA-I* and *M*, lack *toyJKL*, resulting in the exclusive synthesis of compound **15**.

**FIGURE 4:**
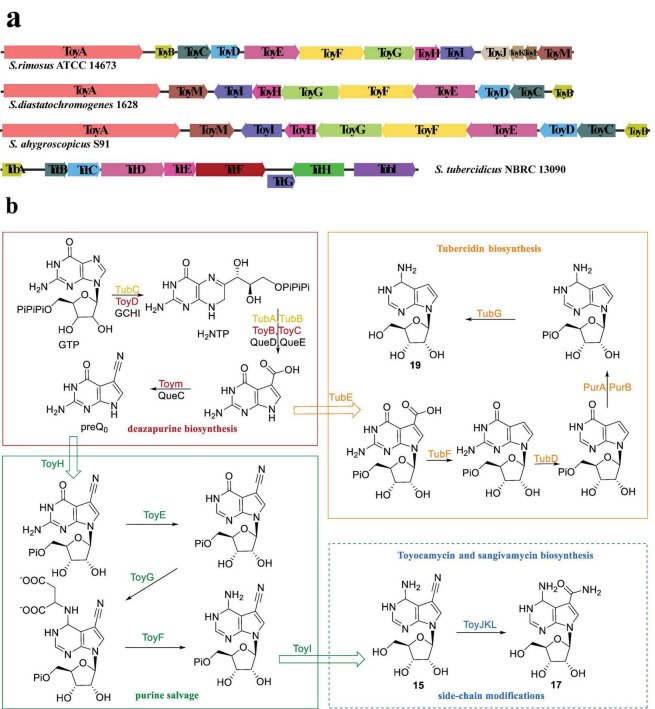
Gene clusters and the biosynthetic pathways of pyrrolopyrimidine nucleosides analogous (Adapted from references: McCarty and Bandarian, 2008; Xu et al., 2019; Liu et al., 2018). **(a)** The toy and tub gene clusters derived from *S. rimosus* ATCC14673, *S. diastatochromogenes* 1628, *S. ahygroscopocus* S91, and *S. tubercidicus* NBRC13090, respectively, where genes of the same color represent homologous functions. **(b)** Color-coded functions in the biosynthetic pathway; red box denotes the deazapurin biosynthesis stage in the production of compound **15**, green box represents the purine salvage stage, blue dotted box is solely relevant to the biosynthesis of **17**, orange box is associated with the biosynthesis of **19**, and the enzymes marked black are involved in PreQ0 biosynthesis.

McCarty and Bandarian (2008) conducted a study on the biosynthesis pathway of the *toyA-M* gene cluster in *S. rimosus* ATCC 14673. According to the synthesis process, the 13 genes are categorized into three catalytic steps. Specifically, genes *toyB, C, D*, and *M* encode enzymes for deazapurin synthesis, while genes *toyE-I* encode enzymes for purine salvage (Figure 4b; McCarty and Bandarian, 2008). It is worth noting that genes *toyJ-L* encode Toyocamycin nitrile hydratase (TNHase), which play a role in the third step involving the hydration of cyanide-containing compounds from **15** to **17**. However, these three genes are solely associated with the synthesis of compound **17**. Consequently, in *S. diastatochromogenes* and *S. albulus*, only genes *toyA-I* and *M* are present. The organization and sequence of the *toy* cluster exhibited variations among the three bacterial stains (Figure 4a; Xu et al., 2019). Notably, TNHase distinguishes itself from other nitrile hydratase (NHase) enzymes by comprising three subunits, as opposed to the typical two subunits (Kobayashi et al., 1992). The regulatory gene *toyA*, which encodes a substantial ATP-binding regulator belonging to the LuxR family (LAL-family), plays an essential role in the synthesis of **15**. Disruption of the *toyA* gene led to a near-complete abolished **15** production.

Compound **19** has been recognized for its biological activity for many years, and recent studies have revealed additional bioactivities. Its notable *in vitro* inhibitory activity against drug-resistant strains of *Mycobacterium tuberculosis* and reference strains of no tuberculosis *Mycobacteria* renders it a valuable candidate for further investigation as a potential treatment for mycobacterial infections (Sun et al., 2023). Additionally, the compound has exhibited significant antiviral activity against both classical and variant strains of porcine epidemic diarrhea virus (PEDV) (Wang et al., 2024). Furthermore, compound **19** exhibited substantial anti-small cell lung cancer (SCLC) activity both *in vitro* and *in vivo*, while demonstrating minimal or negligible cytotoxic effects on normal primary/tracheal bronchial epithelial cells (PBTECs), thus indicating its high selectivity toward SCLC cells and its potential suitability for targeted drug development (Chen et al., 2022). Additionally, the efficacy of **19** and its chemically synthesized analogue **20** has been demonstrated in the treatment of life-threatening conditions such as African trypanosomiasis caused by *Trypanosoma brucei* parasites, Chagas disease, and sleeping sickness (Hulpia et al., 2019a,b; Aldfer et al., 2022).

The biosynthetic pathway of compound **19** was characterized through the reconstruction of the *tub* cluster from *S. tubercidicus* NBRC 13090 in a heterologous host, as depicted in Figure 4a (Liu et al., 2018). Similar to the biosynthetic pathways of **15** and **17**, the first step in biosynthesis of **19** is the conversion of GTP to CDG (7-carboxy-7-deazaguanine), as shown in Figure 4b (McCarty and Bandarian, 2008; McCarty and Bandarian, 2012). In both toyocamycin and tubercidin biosynthesis, 7-carboxy-7-deazaguanine (preQ0) serves as a key intermediate linking the common deazapurine scaffold formation to subsequent tailoring reactions. The functions of TubA-E and G exhibit similarities to those of ToyB-E, H, and I. TubF has been confirmed as an atypical decarboxylase that likely utilizes prenylated-FMN as a cofactor, while the functions of ToyH and I have yet to be verified (Liu et al., 2018).

### 2.4 *N*-NAs without Identified BGCs

Additional compounds, including 3′-amino-3′-deoxyadenosine (**21**) (Pugh and Gerber, 1963), 2′-amino-2′-deoxyadenosine (**22**) (Gao et al., 2017), 2′-amino-2′-deoxyguanosine (**23**), neplanocin B (**24**), neplanocin C (**25**), neplanocin F (**26**) (De Clercq, 1985; Hamon et al., 2010) and cadeguomycin (**27**) (Yuan et al., 1985), are also classified as purine-derived *N*-NAs (Figure 1).

Among these compounds, **21**, **22**, and **27** have been reported to inhibit tumor growth and metastasis, demonstrating clear antitumor activity (Pugh and Gerber, 1963; Shigeura, 1975; Nakanishi et al., 1976; Sato et al., 1979; Tanaka, 1983; Yuan et al., 1985). Compounds **24**–**26** were obtained from the soil fungus *Ampullariella regularis* along with **8** and **9**. Compound **8**, which serves as a lead compound in the neplanocins family, has demonstrated antiviral effects, and both **8** and **26** exhibit antitumor activities in mice (Hayashi et al., 1980; De Clercq, 1985; Hamon et al., 2010). However, the majority of existing research on the aforementioned compounds primarily examines their biological activity and chemical synthesis, with no literature reporting their biosynthetic pathways. However, plausible probes can be proposed based on their structural features and likely biosynthetic logic. These compounds share purine-based scaffolds and exhibit aminotransferase- or dehydrogenase-dependent modifications similar to those found in pentostatin- and angustmycin-type pathways. Therefore, genes encoding adenosine deaminase like enzymes, ribose 5-phosphate pyrophosphokinases, or aminotransferases could serve as potential probes for mining related clusters in actinobacterial or fungal genomes.

## 3 Mining for biosynthetic gene clusters encoding for enzymes responsible for production of purine-derived *N*-nucleoside antibiotics

**FIGURE 5:**
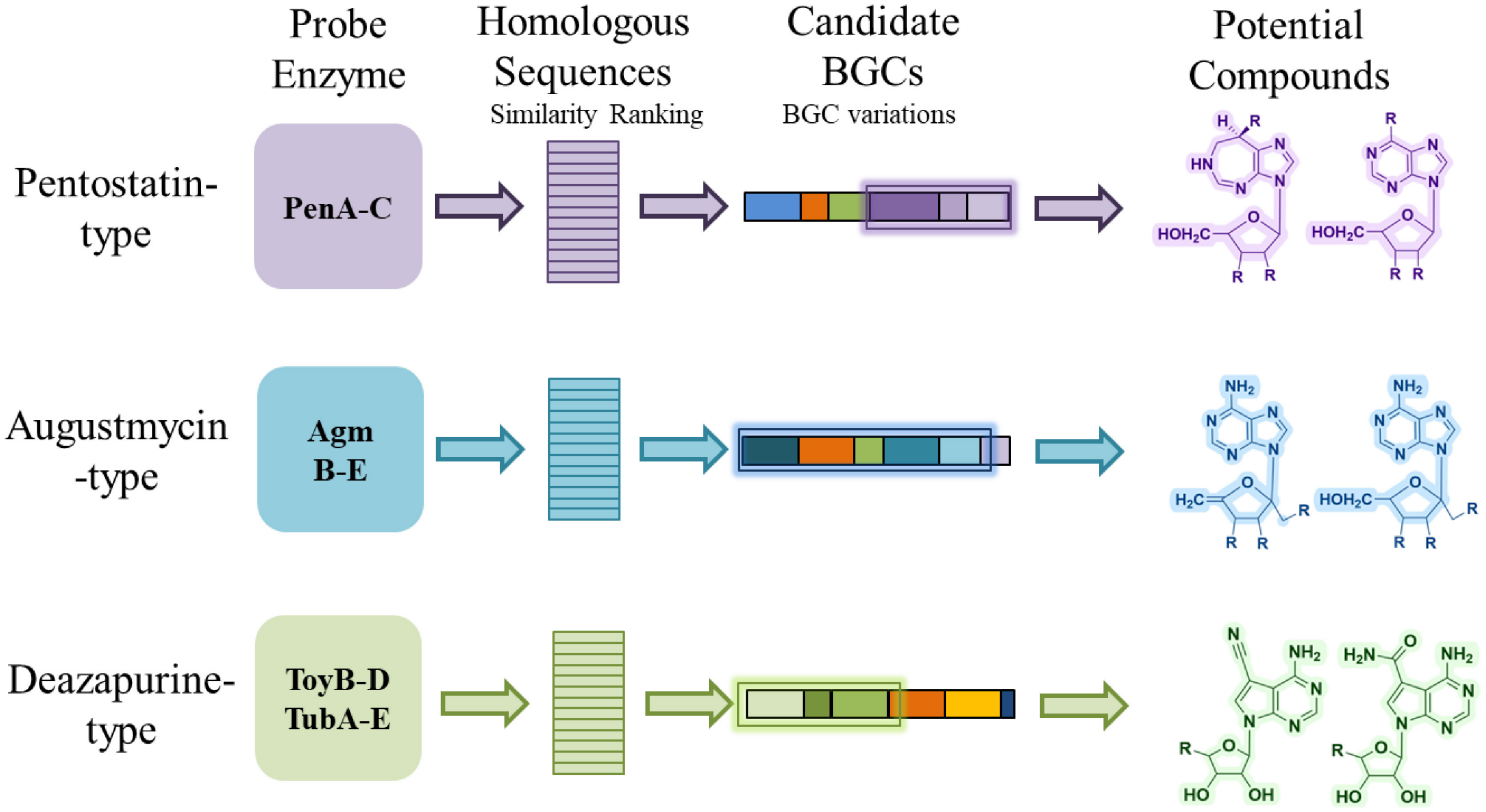
Probe-based genome mining of three types of purine-derived *N*-nucleoside antibiotics.

A widely used approach in genome-guided discovery of natural products is to employ experimentally characterized core enzymes as sequence probes to identify homologous BGCs (Shi et al., 2019; Bauman et al., 2021). For example, the *pen* BGC and the *mac* BGC mentioned in Section “2 Bioactivity and biosynthesis of purine-derived *N*-nucleoside antibiotics” were both identified by using their respective core enzyme as sequence probes (Wu et al., 2017; Xu et al., 2018). This strategy has been successfully applied to various classes of secondary metabolites and is increasingly used for nucleoside antibiotics (Du et al., 2023). By focusing on enzymes that are essential for scaffold construction and contain conserved catalytic motifs, researchers can retrieve candidate BGCs from genome databases and generate hypotheses about the possible products encoded by these clusters (Figure 5).

It is noteworthy that most purine-derived *N*-NA biosynthetic gene clusters cannot be predicted by antiSMASH, and only one cluster among the reported examples is recognized, emphasizing the need for probe-based genome mining approaches. To illustrate this concept, we summarize three representative groups of purine-derived *N*-NAs and the enzymes most commonly used as probes. For PTN-type compounds, enzymes such as PenABC play a crucial role in the synthesis of PTN-related compounds. These proteins are highly conserved and widely distributed, making them potential candidates for identifying new PTN-related biosynthetic pathways in existing microbial genomes (Wu et al., 2017). For AGMs, the minimal conserved set AgmB-E was employed, and retrieved sequences may suggest variants that differ from known AGMs by the presence or absence of specific tailoring steps (e.g., absence of AgmF may be linked to bacterial adaptation to the surrounding environment, and the lack of AgmA allows for the utilization of adenine as a substrate for AGM production in certain bacterial strains or the potential involvement of alternative phosphoribohydrolases in a manner akin to AgmA, without being confined to a gene cluster) (Mitani et al., 1977; Yu et al., 2021). For deazapurine analogues (e.g., toyocamycin, tubercidin), the biosynthesis of preQ_0_ necessitates the involvement of four enzymes (ToyB-D and M). Subsequently, preQ_0_ is converted to **15** and **17** by ToyE-I (McCarty et al., 2009; Battaglia et al., 2011; Yuan et al., 2018). Therefore, the presence of ToyB-D/TubA-C is essential for the biosynthesis of deazapurines. Additionally, ToyH/TubE and ToyI/TubG perform analogous functions in adenylosuccinate synthesis and dephosphorylation, respectively. Consequently, it can be inferred that a BGC containing enzymes similar to ToyB-D, H, and I may be involved in deazapurines biosynthesis. Therefore, ToyB-D and TubA-E are suitable probes; retrieved hits indicate potential producers of toyocamycin-like scaffolds, though variations in cluster composition may lead to novel analogues distinct from those already characterized.

In this way, the conceptual flow from probe enzyme, through homologous sequence identification and candidate cluster recognition, to predicted compound provides a framework for genome mining.

## 4 Conclusions and perspectives

Purine-derived *N*-NAs constitute a structurally diverse and biologically versatile family of natural products. Their remarkable range of activities–spanning antibacterial, antifungal, antiviral, and antitumor effects–underscores their potential as valuable therapeutic agents. Advances in elucidating the biosynthetic pathways of representative compounds, including PTNs, AGMs, and deazapurine analogues, have revealed a set of conserved enzymatic transformations that underpin scaffold construction and structural diversification.

The increasing availability of microbial genome sequences, coupled with refined genome mining strategies, provides new opportunities to uncover additional *N*-NA BGCs. Core biosynthetic enzymes, once biochemically characterized, can serve as informative probes for identifying homologous clusters in diverse microorganisms. Nevertheless, bioinformatic predictions should be regarded as hypothesis-generating: the functional assignment of putative clusters requires experimental validation through genetic, biochemical, and metabolomic approaches.

Looking forward, several challenges remain. First, the structural complexity of *N*-NAs often involves tailoring steps that are poorly understood, complicating the prediction of final products. Second, the functional redundancy of enzyme homologs across different BGCs increases the difficulty of distinguishing genuine *N*-NA clusters from unrelated pathways. Third, translating genome mining leads into practical drug discovery will require efficient heterologous expression systems and advanced analytical pipelines for metabolite detection.

Despite these challenges, the integration of genomics, synthetic biology, and high-resolution metabolomics is expected to accelerate the discovery of novel *N*-NAs. By linking conserved enzymology with innovative mining approaches, researchers can expand the known chemical space of nucleoside antibiotics and identify promising leads to address the urgent demand for new antimicrobial and anticancer agents.
